# The degradation and detection of environmental signals in sediment transport systems

**DOI:** 10.1126/sciadv.adi8046

**Published:** 2023-11-03

**Authors:** Chloe Griffin, Robert A. Duller, Kyle M. Straub

**Affiliations:** ^1^ Department of Earth, Ocean and Ecological Sciences, University of Liverpool, 4 Brownlow Street, Liverpool L69 3GP, UK.; ^2^ Department of Earth and Environmental Sciences, Tulane University, 6823 St. Charles Avenue, 202 Blessey Hall, New Orleans, LA 80118-5698, USA.

## Abstract

Autogenic processes contribute noise to sediment transport systems that can degrade or mask externally derived environmental signals and hinder our ability to reconstruct past environmental signals from landscapes and strata. To explore this further, we measure efflux from a physical rice pile to ascertain the temporal structure of autogenic noise, and how this influences the degradation and detection of environmental signals. Our results reveal a tripartite temporal spectral structure segmented at two key autogenic time scales. The shorter autogenic time scale set limits on environmental signal degradation, while the longer autogenic time scale sets limits on environmental signal detection. This work establishes a framework that can be used to explore how autogenic processes interact with external environmental signals in field-scale systems to influence their detectability. We anticipate that the temporal structure and associated time scales identified will arise from autogenic processes in numerous sediment transport systems.

## Teaser

A physical avalanching rice pile generates three autogenic noise regimes that govern input signal degradation and detectability.

## INTRODUCTION

Sediment transport systems (STSs) are sensitive to external environmental perturbations; these can be natural (e.g., related to climatic or tectonic processes) or anthropogenic in origin (*1*–*5*). STSs respond and adjust to these perturbations in a number of ways and over a range of temporal and physical scales (*2*, *6*). A fundamental response of a STS to these perturbations is a variation in the generation of sediment supplied to the STS and transmitted down system as an environmental signal (*4*, *7*). These environmental sediment flux signals can generate geomorphic and stratigraphic signatures that allow for the reconstruction of past environmental perturbations (*4*, *7*–*11*) and provide insight into the response of landscapes to future environmental change (*12*, *13*).

However, environmental sediment flux signals can undergo varying degrees of modification during their propagation through STSs and to strata (*14*). This is primarily due to episodes of sediment storage and release that occur along the length of STSs in a stochastic manner and are referred to as autogenic processes (*2*, *4*, *14*, *15*). Even under constant boundary conditions, autogenic processes induce sediment storage and release over a range of spatiotemporal scales (*15*–*23*) from centimeter-scale bedforms migrating over seconds (*24*–*28*) to delta lobes avulsing hundreds of kilometers over millennia (*26*, *29*–*31*). This stochasticity means that a one-to-one correlation between a singular or periodic environmental perturbation and a sedimentary-proxy record for the associated environmental sediment flux signal is not guaranteed (*4*, *14*, *15*, *32*). Autogenic processes are a natural physical phenomenon that are ubiquitous across many landscapes and occur in the absence of external environmental perturbations (*15*, *33*, *34*). Autogenic processes are commonly associated with a self-organized behavior of STSs over sufficiently long time scales (*34*), where the time required for a STS to self-organize is scaled to the size of the system in question and the nature of the interactions between internal system components (*15*). The self-organization of a physical system can be viewed as a statistical property (*35*) and as a measurable property. Examples of the latter include the regular spacing of point bars in meandering rivers (*15*), the size distribution of sediment storage and release events from a time series of sediment flux, or the organization of surface topography and strata (*26*, *36*).

We note that many measurable attributes of STSs follow heavy-tailed distributions that are truncated at the upper end [e.g., the magnitude of erosional and depositional events; (*37*)]. The shape of this distribution is determined by the specific transport mechanisms and depositional dynamics, and the upper truncation is due to the bounding effect of system size that sets a physical limit on the spatiotemporal scales of autogenic processes (*37*). In the broadest sense, self-organization is an emergent property of a system that can be used to make predictions about the overall behavior of a system (*15*, *35*). However, autogenic processes also contribute noise to a STS in the form of autogenic sediment flux or “natural variability in sediment flux” (*2*, *9*, *14*–*16*, *26*, *38*–*40*), which will also impart variability to strata (*6*, *16*, *32*, *41*–*43*). This noise can severely limit the identification of an environmental sediment flux signal either by obscuring it, i.e., the power of autogenic noise is greater than the environmental signal itself (*44*), or by interacting with it to such an extent that no trace remains of the original signal (*6*, *45*, *46*), i.e., the signal is shredded [sensu (*14*)]. These two mechanisms are in operation simultaneously and will act to reduce the detectability of environmental signals from a time series of sediment flux. However, the concepts of signal shredding and detectability have become somewhat intertwined, where all undetectable signals are considered shredded (*7*, *14*, *47*). The relationship between these concepts, and a framework to predict when signals are shredded and/or undetectable, is not yet established. To do this, we use a physical rice pile as a rudimentary and idealized STS. Rice piles have previously been shown to exhibit a complex behavior (*48*, *49*) and are drawn upon to understand autogenic processes and environmental signal propagation through STSs (*14*). The aim here is to characterize the full temporal structure of autogenic noise and associated time scales from a physical model and to understand how the periodicity and amplitude of imposed environmental signal interacts with the autogenic noise. This will provide a robust theoretical framework that can be used as a starting point to explore the autogenic temporal structure of field-scale STSs and how this information can be used to generate confidence limits of environmental signal detectability and thresholds of signal shredding (*15*) in STSs and associated strata. More broadly, this is crucial to the accurate reconstruction of past environmental signals and to our ability to predict how environmental signals will interact with STSs over a range of time scales to garner a detectable (or not) response.

### Theoretical background

The time scale required for the largest landscape components of STS (e.g., rivers or delta systems) to self-organize is beyond the time scales of human observation and modern instrumental records (*50*), and so field-scale systems are unsuitable targets to fully characterize the autogenic structure of STSs and the interaction of autogenic processes with environmental signals. To overcome this, physical experiments and numerical models are used (*50*). One such numerical experiment, and pertinent here, is a numerical one-dimensional (1D) avalanching rice pile, which has offered key insights into the structure of stochastic noise (*51*) and the role of autogenic processes in environmental signal shredding (*14*). The 1D numerical rice pile models, although rudimentary, elucidate the nature of autogenic processes and provide a basis from which natural STSs and strata can be understood (*6*, *15*, *33*), especially with regard to environmental signal shredding and detectability (*14*). Although these models capture the nature of stochastic dynamics well, one drawback is that they rely on user-defined thresholds to control the propagation of individual particles through the model domain rather than natural physical thresholds (*48*). In addition, numerical rice pile models do not allow for transport of grains out of the model domain without experiencing storage on the surface and contributing to the construction of topography until a critical angle is exceeded whereby an avalanche occurs. In natural systems, sediment has the capacity to propagate through a system with minimal storage or deposition and which could enhance propagation and detection (*40*, *52*). Physical 1D rice piles do not suffer from these limitations and offer a richer suite of autogenic statistics that arises from sediment storage and release along a 1D transport path, analogous to sediment transport in a 2D path in field-scale systems (*14*, *49*).

Jerolmack and Paola (*14*) determined the structure and time scales of autogenic noise using a time series of efflux generated from a numerical avalanching rice pile model and proposed a framework for the propagation and storage of environmental signals. In their study, the structure of autogenic noise was found to exhibit two regimes. The first regime comprises temporal correlation (red noise) over short time scales, where spectral power increases as a function of period. The second regime comprises zero correlation (white noise) over all succeeding time scales, where the spectral power plateaus. The transition between the red noise and white noise regimes denotes a characteristic time scale *T*_x_, which was hypothesized to scale as ~*L*^2^/*q*_0_, where *L* is system length and *q*_0_ is input rate. *T*_x_ represents the upper temporal limit on the ability of autogenic processes to “shred” environmental signals (*14*). Environmental signals with periods greater than *T*_x_ are recorded in the discrete time power spectral density of the efflux (hereafter called power spectra), whereas those with periods less than *T*_x_ are shredded as the periodicity of the input signal is within the scale of individual sediment transport events in the system, obliterating evidence of the signal (*14*).

While the presence of white noise in STS is expected to persist over all time scales greater than *T*_x_ (*14*), the results of other numerical sandpile models find the presence of blue noise (anticorrelation) over the longest time scales (*51*, *53*), where spectral power decreases as a function of period. The presence of anticorrelation within STS is due to the size constraints of a system, which places an upper limit on the size of the largest sediment transport event (finite size effects). This finite size effect is reflected by a gradient break in the resulting power spectra at the transition from white noise to blue noise (*37*, *51*, *54*–*56*). Within the correlated regime (red noise), the system continues to operate in the same way as the previous time step (e.g., stabilization of channel networks on a delta, which allow the system to generate consistently high sediment fluxes). However, anticorrelation relates to a behavior where the largest events are always followed by small events as the system regenerates or regrades over these longer time scales (*15*). Anticorrelation or blue noise is common in power spectra from numerical sand and rice piles (*51*, *53*), ecological models (*57*), ice-core analysis (*58*), and population dynamics (*59*), hinting at a universal structure due to the finite size behavior of stochastic systems over their longest time scales.

In the same manner as *T*_x_ was defined by spectral gradient breaks (*14*), the spectral gradient break from white noise to blue noise denotes the presence of another autogenic time scale, which was suggested to scale with a system-wide discharge event (~*L*^2^) [*T_c_* of Hwa and Kardar (*51*)]. The numerical rice pile investigations of both Hwa and Kardar (*51*) and Jerolmack and Paola (*14*) report a short autogenic time scale (i.e., transition from red noise to white noise), but a discrepancy exists in the definition and scaling of this fundamental time scale. Hwa and Kardar (*51*) suggest that the transition from red noise to white noise scales as the maximum duration of avalanches [*T*_a_ of Hwa and Kardar (*51*)], while Jerolmack and Paola (*14*) suggest that this time scale represents a wedge-filling time scale on the order of *L*^2^ [*T*_x_ of Jerolmack and Paola (*14*)]. The latter definition overlaps somewhat with the definition of the longer autogenic time scale by Hwa and Kardar (*51*).

**Fig. 1.**
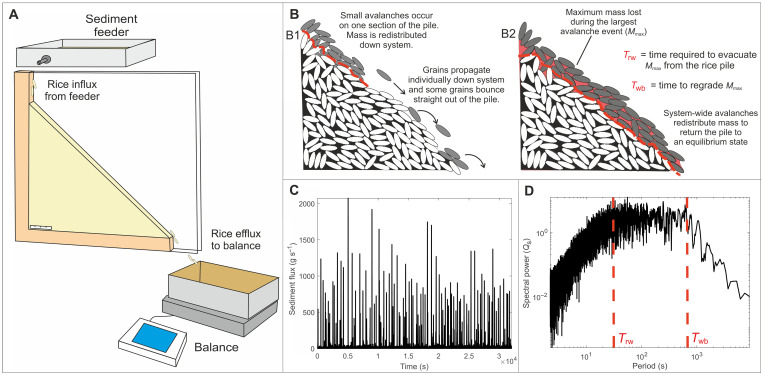
The geometry and nature of rice pile experiments. (**A**) Schematic diagram of the experimental rice pile set-up. (**B**) Spatiotemporal scales of avalanches within the rice pile, over short time scales (B1), individual grains and small avalanches dominate the time series, whereas over long time scales (B2), avalanches on the order of system size occur. (**C**) Time series of efflux from the physical rice pile run under constant influx rate. (**D**) Power spectra generated from the efflux time series. Autogenic time scales are defined according to spectral gradient breaks.

The structure of autogenic processes (*51*) and the original framework for signal shredding (*14*) is yet to be duplicated within a physical rice pile that evolves under gravity and hence is more comparable to natural STSs. The physical rice pile is analogous to a single sediment routing system (*60*), and the associated temporal structure and time scales of autogenic processes incorporates all of the autogenic variability that this single sediment routing system can offer. The analogy of a rice pile as a single sediment routing system is therefore a simple one but still offers a crucial insight into the autogenic dynamics of natural systems and their ability to shred or transmit environmental signals. Here, we set out to clarify the origin and scaling of these autogenic time scales by resolving the temporal structure of autogenic processes using a 1D physical rice pile. To do this, we use a time series of efflux from the rice pile at discrete time intervals (Fig. 1). This efflux time series is generated from stochastic avalanche dynamics within the rice pile and is a proxy for the autogenic dynamics operating within Earth’s surface. We begin by characterizing the structure and time scales of autogenic dynamics within a system run under constant input rate and use this to understand the controls on signal shredding by imposing signals with periodicity over the full range of autogenic time scales.

## RESULTS

### The temporal structure of autogenic processes

**Fig. 2.**
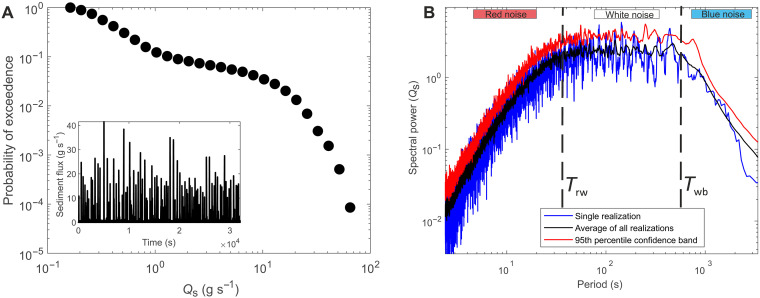
Time series analysis of mass efflux from the control experiment, where influx rate is 0.37 g s^−1^. (**A**) Distribution of avalanche sizes throughout the time series, where the probability shows a heavy-tailed distribution. (**B**) Power spectra of the time series for one realization of the control experiment, generated by the multi-taper method, showing tripartite geometry composed of red, white, and blue noise. Spectral gradient breaks between the regimes mark two time scales: *T*_rw_ and *T*_wb_. This spectrum is compared to the mean spectra from all 25 realizations of the control experiment, with the 95% confidence band generated from the realizations displayed.

To understand how autogenic processes control signal propagation, we must first understand the inherent structure of autogenic processes and quantify the key autogenic time scales intrinsic to the physical rice pile. To do this, we use a time series of efflux measured at discrete time intervals generated from multiple realizations of the control experiment (run under a constant feed rate of 0.37 g s^−1^; ~18.5 grains s^−1^). Constant influx to the physical rice pile generates a range of avalanche event sizes, from continuous small efflux events (e.g., 0.1 g s^−1^; ~5 grains s^−1^) to avalanches that span the entire length of the system (33 to 43 g s^−1^; ~1650 to 2150 grains s^−1^). The wide range of avalanche sizes that occur within the pile are generated from the pile fluctuating around a stationary critical state where localized, individual granular interactions can induce events of system scale. The probability distribution of these avalanches throughout the time series is heavy-tailed (Fig. 2A), meaning that although the time series is dominated by small events (e.g., Fig. 1B1), an avalanche on the order of system size (e.g., a wedge failure event that returns the system to dynamic equilibrium; Fig. 1B2) has a small chance of occurring (*37*). The rich stochastic dynamics evident in the output from the physical system agree with the structure of the internal dynamics observed in numerical models (*14*, *48*, *49*, *61*).

The power spectra generated from the efflux time series from the constant influx experiment exhibits three noise regimes defined by two distinct changes in the gradient of the power spectra (Fig. 2B). The first regime comprises red noise (temporal correlation), whereby spectral power increases as a function of period [with a spectral gradient (α) of 2.2]. The upper temporal limit of red noise denotes a characteristic autogenic time scale (*T*_rw_), which is approximately 30 s for this experiment. The second regime comprises white noise, which occurs over 30 to 650 s, where spectral power plateaus, indicating events over this time scale, are temporally uncorrelated. The upper temporal limit of white noise denotes a characteristic autogenic time scale (*T*_wb_), which occurs at approximately 650 s for this experiment. The third regime comprises blue noise over time scales greater than 650 s, whereby spectral power decreases as a function of period [with a spectral gradient (α) of −2], exhibiting anticorrelation in efflux.

**Fig. 3.**
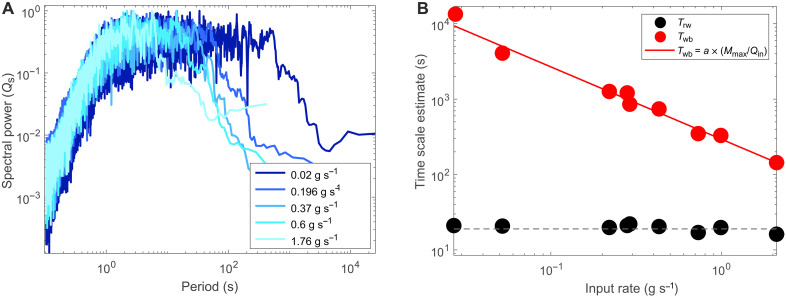
Time series analysis of efflux from four experiments compared to the control experiment. (**A**) Power spectra of the time series, normalized by the mean spectral power from each experiment. Period is normalized by *T*_rw_ (~30 s). (**B**) Comparison of time scales *T*_rw_ and *T*_wb_ with changing input rate, where *T*_rw_ remains constant and *T*_wb_ decreases as a function of input rate. The dashed line shows the line of best fit for the variation in *T*_rw_. The solid red line shows the fit of the equation *T*_wb_ = *a* × (*M*_max_/*Q*_in_).

These three noise regimes exist within the power spectra regardless of the absolute influx rate (*Q*_in_) (Fig. 3A). However, we explore the controls on the absolute spectral duration of each regime and both autogenic time scales, *T*_rw_ and *T*_wb,_ using a suite of experiments run under a range of constant influx rates (table S1). First, we find that the red noise regime and the value of *T*_rw_ are insensitive to the influx rate and remain at a constant value of 30 s. In numerical sandpiles, this spectral regime was found to record the duration of individual avalanche events, where the duration of individual avalanches is directly proportional to avalanche size (e.g., mass effluxed). These individual events increase in duration until an upper cutoff time is reached [*T*_a_ of Hwa and Kardar (*51*) an *T*_rw_ this study], which defines the maximum duration of an avalanche within the system and corresponds to the largest avalanche in terms of total mass liberated (*53*, *58*). Through examination of the efflux time series (fig. S1), we confirm this to also be the case for the physical rice pile. The constancy of the value of *T*_rw_ reflects the fixed dimensions of the system and material properties of the rice material, which fixes the critical angle of repose and therefore sets the duration of the longest avalanche regardless of the influx rate. *T*_rw_ will vary between systems of different lengths (*51*). Over time scales greater than that of individual avalanche events (e.g., the white noise regime), avalanche of all sizes and duration coalesce, increasing the duration over which efflux occurs (*53*, *58*). In other words, the onset of one avalanche can instigate another avalanche, and so the efflux measured is the result of merged events. *T*_wb_ on the other hand, which sets the upper limit to the white noise regime, is influx rate dependent (Fig. 3B). In numerical sandpiles, this longer time scale was suggested to scale with *L*^2^ and influx rate; however, the precise dependence was not determined (*36*, *53*, *58*). We find that *T*_wb_ represents the time required for the influx to regrade the mass lost in the largest avalanche event (a regeneration time scale). The value of *T*_wb_ can be predicted by *T*_wb_ = *a* × (*M*_max_/*Q*_in_), where *M*_max_ is the maximum mass efflux over the longest avalanche event (defined by *T*_rw_) and *a* is a parameter value that accounts for a bypass fraction of the efflux as the pile regrades; this is required as the rice pile is an open system, and hence, efflux still occurs while the mass regrades. Here, *a* has a value of 1.38 ± 0.13 (*n* = 8). For this experiment, *M*_max_ is approximately 142 g (fig. S1); this will be discussed later. Over time scales greater than *T*_wb_, the rice pile experiences avalanches that are of the order of system size, which return the pile from the maximum to the minimum slope.

### Shredding and detection of environmental signals

**Fig. 4.**
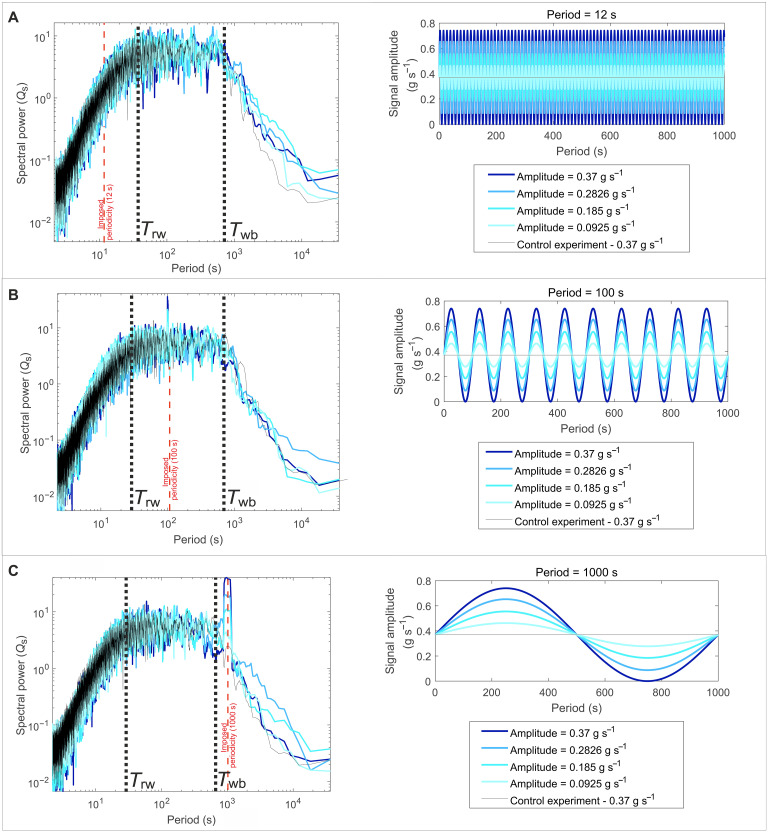
Power spectra generated from a suite of rice pile experiments with imposed signals in the form of cyclic rice influx. Spikes in power at the imposed periodicity highlight the presence of imposed signals. The power of the signal spike decreases as signal amplitude decreases. Each panel contains five power spectra, 4 from rice pile experiments with imposed periodicity where the imposed periodicity is constant but signal amplitude decreases in reference to the mean feed rate and also the spectra from the control experiment. (**A**) Imposed periodicity of 12 s. (**B**) Imposed periodicity of 100 s. (**C**) Imposed periodicity of 1000s. We highlight that spectral structure is not influenced by the addition of external forcing. The imposed influx signals are shown in relation to both autogenic time scales (*T*_rw_ and *T*_wb_) by the dashed red lines.

Given that autogenic processes can alter environmental signals, we explore how the sediment transport mechanics associated with each spectral noise regime control signal propagation, and hence how both autogenic time scales (*T*_rw_ and *T*_wb_) set thresholds for signal shredding and signal detection. We define shredded environmental signals as those signals that have undergone a severe reduction in amplitude during propagation through the rice pile. From now on, these will be referred to as degraded signals. We define detectable environmental signals as those signals that produce a spectral peak within a power spectra that exceed the range of autogenic noise; this is defined statistically by the 95% confidence band. These concepts are defined separately as they describe different properties of environmental signals, but we emphasize that they do not always coincide, e.g., degradation does not define detectability. To understand thresholds for signal degradation and detection, we ran a suite of physical rice pile experiments with imposed sediment influx signals. The periodicities of the signals spanned the full temporal range of autogenic time scales, from below *T*_rw_ to above *T*_wb_ (Fig. 4), to delimit the influence of both autogenic time scales. Furthermore, to understand the effect of signal amplitude on signal degradation and detectability, we systematically varied the signal amplitude for each periodicity. For parity with the control experiment, all the imposed signals share the same mean feed rate (0.37 g s^−1^) but decrease in amplitude from 100 to 25% of the mean feed rate.

To quantify degradation, we require knowledge of the amplitude of the output signal relative to the known input signal. Here, degradation is comparable to the concept of “gain” used to analyze the propagation and preservation of environmental signals within diffusive systems [e.g., (*62*)].

To quantify the output signal amplitude, the efflux time series from an experiment with imposed periodicity is first divided into lengths equal to the input period. Then, the mean efflux is taken every second over the input signal period; this mean efflux time series should approximately resemble the input signal. To this mean efflux, we then fit a sine wave where the period is predefined (the known input periodicity), but the amplitude and phase shift are returned depending on the output signal identified. We compare the amplitude of the signal evident in the output flux to that of the known input signal and quantify a percentage similarity (fig. S2).

**Fig. 5.**
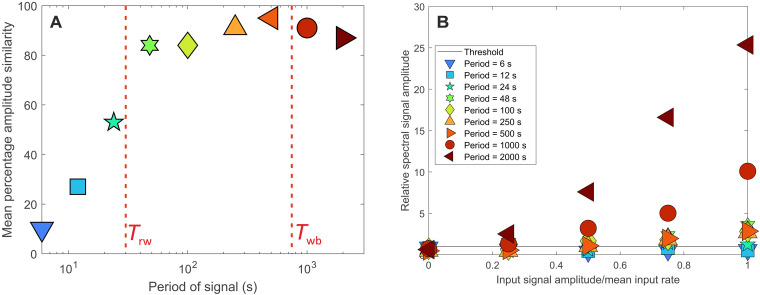
Degradation and detectability of environmental signals. (**A**) Signal degradation as a function of input period, measured by comparing the known input signal, to the signal evident in the efflux from stacking multiple realizations (see Materials and Methods). (**B**) Signal detectability as a function of both input period and amplitude. Power of the signal spike at the imposed periodicity compared to the power of the 95% confidence band at the imposed periodicity. The data at 0 amplitude represent an experimental run with no imposed periodicity. *Y* axis data points are calculated as power at imposed period/power of confidence band at imposed period; hence, values greater than 1 breech the confidence band.

We find that input signal periodicity is the primary control on signal degradation and that the short autogenic time scale, *T*_rw_, sets an upper limit to the time scales over which signals experience degradation (Fig. 5A). Over all periodicities, signal amplitude does not influence the amount of signal degradation. Signals with periodicity less than *T*_rw_ experience severe degradation, where the smaller the periodicity of the signal, the greater the amount of degradation experienced. We highlight these signals are not completely destroyed [i.e., shredded (*14*)] but are reduced in amplitude. In comparison, signals with periodicities greater than both *T*_rw_ and *T*_wb_ experience minimal degradation, where the output signal exhibits on average 90% similarity to the known input signal over all periodicities greater than *T*_rw_ (Fig. 5A). We note that signal amplitude does not influence the amount of degradation a signal experiences; signals of the same periodicity are degraded by equal amounts regardless of their input amplitude (fig. S3). However, we acknowledge this may not be the case for signals with larger amplitudes (e.g., those on the order of *M*_max_).

We also explore the relationship between the degradation of input signals and their detectability. To make a statistical statement about the presence of an influx signal within the power spectra, a confidence band was generated from the background structure of autogenic processes, which allows autogenic noise to be differentiated from imposed periodicity. We generate a 95th percentile confidence band from a suite of control experiments (Fig. 2B), all sharing the same forcing conditions, by calculating the percentage of the power spectra that falls above a given power for each period. To quantify detectability, we compare the spectral power of the signal spike to the spectral power of the 95% confidence band at the imposed periodicity; detectable signals are those that breach the confidence band. We find that signals with periodicity less than *T*_rw_ do not generate a spectral response that exceeds the 95% confidence band and so are statistically undetectable in the output flux, regardless of amplitude (Fig. 5B). We acknowledge that this may not be the case for signals with larger amplitudes (e.g., those on the order of *M*_max_). Above *T*_rw_, signal amplitude influences the detectability of signals; the greater the amplitude of the signal, the greater the ratio of the spectral peak to the confidence band. Large amplitude signals with periodicity between *T*_rw_ and *T*_wb_ are easily detectable in the output flux, but small amplitude input signals can be rendered undetectable in the output flux. This is because the amplitude of the signal is of the same magnitude as autogenic fluctuations, i.e., the signals are obscured by autogenic noise. However, above *T*_wb_, the amplitude of the resultant signal spike is much greater than that of the confidence showing enhanced detectability. Signals with periodicity over these long time scales are greater than the largest autogenic fluctuations and therefore overwhelm the noise produced by autogenic processes. Therefore, *T*_wb_ sets a temporal threshold beyond which the detectability of environmental signals is enhanced.

## DISCUSSION

### Separating thresholds for the shredding and detection of environmental signals

Our physical experiments show the presence of a short autogenic time scale, *T*_rw_, denoting the red noise to white noise transition in the power spectra. *T*_rw_ in the physical rice pile is analogous to *T*_x_ found in numerical rice pile systems (*14*), and our experiments confirm that *T*_rw_ provides an upper limit to the time scales over which signals experience shredding. Jerolmack and Paola (*14*) found that short period input signals (*T* < *T*_x_), with amplitudes below an exceedance that would otherwise induce system clearing events (*14*), were not detectable in the power spectra and were described as completely obliterated (i.e., shredded). However, we show that small amplitude influx signals can be reconstructed by stacking the output flux if the periodicity is known, suggesting that input signals are not obliterated but rather severely degraded in amplitude. Small amplitude influx signals are of similar magnitude to autogenic fluctuations within STS, so storage and release processes can physically smear short period input signals out over a band of time [i.e., signal shredding (*14*)], which consequently reduces the amplitude of an input signal (degradation) and hence the power of the input periodicity. We modify the original definition of signal shredding to: the smearing of externally-driven signals by sediment transport processes across a range of spatiotemporal scales, resulting in the amplitude of the environmental signal at the system output being severely degraded when compared to the amplitude of the original signal. Although *T*_x_ provides a threshold for shredding in the numerical rice pile, Jerolmack and Paola (*14*) did find that a separate threshold existed for the detectability of shredded signals, where the signal produced a measurable response in the power spectra. They found that only signals with periodicity *T*/*T*_x_ < 0.6 are rendered undetectable in the output flux, whereas the output flux showed evidence of periodicity when the signal periodicity was *T*/*T*_x_ *=* 0.6 to 1. Our physical experiments show this is not the case. We find that all signals with periodicity less than *T*_rw_ are rendered undetectable in the output flux, and hence, *T*_rw_ does provides an upper limit for signal degradation and a lower limit for signal detectability.

We also find that signals with periodicity greater than *T*_rw_ can be rendered undetectable if obscured by autogenic noise (*44*). This finding augments earlier work that hypothesized that the red noise to white noise transition acts as “a lower limit on the ability to pass and record environmental signals” (*14*). Instead, we find that signal detectability is amplitude dependent at time scales between *T*_rw_ and *T*_wb_. We show that only at time scales greater than *T*_wb,_ is faithful signal transfer expected over all amplitudes, as the signal period is greater than the longest time scale autogenic process. Therefore, we find that it is the truncation time scale *T*_wb_, not *T*_rw_ (*T*_x_) that is “the lower limit for the faithful propagation and recording of environmental signals within landscapes,” a definition originally given to *T_x_* (*14*). This is also found to be the case for theoretical frameworks defining signal detectability for longer time scale stratigraphic analysis (*6*, *33*).

Jerolmack and Paola (*14*) considered *T*_x_ (here *T*_rw_) to be comparable to the “basin filling time scale” or the “equilibrium time scale” (*T*_eq_) in a deterministic diffusional framework of landscapes, representing the time required to completely regrade surface topography to a steady state following a perturbation (*63*). However, we suggest that *T*_eq_ is more appropriately approximated by the longer autogenic time scale *T*_wb_, which comes about through the shared property of complete surface regrading or topographic filling, that takes place over these time scales. While *T*_wb_ and *T*_eq_ are comparable regeneration time scales, *T*_rw_ and *T*_eq_ both denote upper limits to the time scales over which environmental signals experience degradation; signals propagating through a diffusional system do not experience a reduction in amplitude (“gain”) when the signal period considerably exceeds *T*_eq_ (*64*). However, the time scales over which signal degradation occurs in stochastic systems (<*T*_rw_) could be up to an order of magnitude less than within diffusional systems (if *T*_wb_ is approximately equal to *T*_eq_), but this is dependent on the mechanics of sediment transport within the system and the influx rate to the system, which governs the separation of *T*_rw_ and *T*_wb_. The reason for this difference in behavior is that a periodic sediment supply signal will pass unimpeded through a diffusive system (i.e., no degradation) once a system-wide topographic steady state is achieved (*4*), whereas in stochastic systems, the signal period must only exceed that of the largest autogenic event. As autogenic processes have no role within a diffusion framework due to the averaging of lateral stochastic system dynamics, *T*_rw_ does not exist and signals can therefore only be related to *T*_eq_. This leads to a loss of predictive capability when evaluating the limits of environmental signal propagation across Earth’s surface, as only long time scale signals can be assessed (*65*). As autogenic processes are inherent to 3D STS and set a lower limit for signal propagation and preservation, any theoretical framework must incorporate stochastic dynamics.

### The detectability of shredded signals

At time scales less than *T*_rw_, we find signal amplitude to have no effect on the degree of signal degradation, but we acknowledge that a threshold must exist within the system beyond which high-amplitude short-period input signals are detectable in the output flux. This amplitude or magnitude threshold (*M*) is expected to scale with the maximum size of autogenic events in the numerical rice pile, i.e., *M* ~ *L*^2^*S*_c_, where *S*_c_ is the critical threshold slope (*14*). In the numerical rice pile, *M* represents the maximum volume of rice effluxed over the longest avalanche event (analogous to *M*_max_ in this study). *M* was defined on the basis that the short autogenic time scale scaled with sediment flux [e.g., a volume filling time scale, equivalent to *T*_eq_; (*14*)], which we show not to be the case for *T*_rw,_ but instead applies to the longer autogenic time scale, *T*_wb_. Therefore, we postulate that the amplitude required for a degraded influx signal to be detectable is much lower than *M*, as the signal amplitude is only required to exceed that of individual autogenic events, rather than the mass required to achieve a system-wide topographic steady state. Furthermore, in the numerical rice pile, the model does not allow for grains to propagate through the model without experiencing storage (analogous to washload sediment in rivers). In this model, grains that enter the model at the top of the pile instantly “stick” at the input location and remain in the model unless liberated by an avalanche. However, in the physical rice pile (and STS), grains have the ability to propagate through the system with minimal storage. Therefore, the inclusion of suspended and/or washload sediment increases the efficiency of signal propagation. These reasons allow us to anticipate that amplitude required for the detectability of degraded signals is much lower than *M*.

The amplitude of the largest signals *T < T*_rw_ imposed onto the physical rice pile is equal to the mean feed rate, with a total influx much lower than *M*_max_, and hence are severely degraded and undetectable in the output flux. However, we suggest that if the signal amplitude exceeded the mean feed rate, or the rate at which the influx rate varied was increased, the signal would be degraded by the same amount but would be detectable in the output flux. A square wave input signal with periodicity less than *T*_rw_ and an amplitude equal to the mean feed rate was imposed onto the physical rice pile and produced a detectable response in the output flux (fig. S4). We hypothesize this to be the case as the total mass influx of a square wave signal is greater than that of a sine wave signal over the same periodicity. This means that a signal can be both severely degraded in amplitude, but the spectral spike can exceed the 95% confidence band. Once the amplitude of the signal is equal to or greater than *M*_max_, these signals will overwhelm the magnitude of the autogenic processes, and hence, we hypothesize that these signals will pass through the system without experiencing degradation. A pathway for future work will be to quantify the amplitude threshold over which short-period signals experience no degradation and explore the nature of this threshold as a function of input periodicity.

While quantifying the effects of autogenic processes is important for understanding signal shredding, we note that quantifying the detectability of signals over all periodicities in landscapes and sedimentary layers takes precedence when reconstructing past environmental signals from landscapes and strata. Power spectra are the most common method used to search for evidence of environmental signals from a time series of stratigraphic measurables. However, the use of power spectra alone is insufficient if signals have been rendered undetectable due to degradation/obscuring by autogenic noise. In this case, the only way to truly show the presence of external signals is if one knows for certain the periodicity and can stack multiple realizations of the signal to reconstruct it. However, when working with time series generated from stratigraphic measurables, the messy conversion of space to time (e.g., the assumption of linear sedimentation rate) brings substantial error into a reconstructed time series over meso-time scales (~10^1^ to 10^5^ years) (*4*, *32*, *66*, *67*). This, alongside the lack of knowledge of the imposed periodicity to search for, makes this methodology generally unfeasible, and hence, we have to rely on power spectra. If environmental signals could be identified in a time series without the use of power spectra (e.g., the time series has excellent age control so the signal could be reconstructed by stacking the time series), then this would remove the requirement to define appropriate statistical thresholds (i.e., confidence levels) to differentiate signal from noise in power spectra. This is beneficial, as the application of ill-fitting thresholds can generate false positives and promote misinterpretations regarding the presence of periodicity (*68*). The identification of environmental signals from power spectra can be aided by knowledge of key autogenic time scales, such as those presented here. For example, the interaction between an environmental signal of known periodicity and autogenic processes can guide scientific practitioners as to whether a signal is not detectable due to shredding (*T* < *T*_rw_) or whether the signal has been obscured by autogenic noise (*T > T*_rw_).

### Rice piles to landscapes to strata

The specific sediment transport and storage mechanisms within an STS will determine the nature and time scales of autogenic processes, which mediates how STSs might transmit environmental signals (*15*, *33*, *65*). In the physical rice pile, the temporal extent of correlation (i.e., red noise up to *T*_rw_) is defined by the duration of individual avalanche events. The rice pile is analogous to an individual component or segment of a STS, such as a hillslope experiencing sediment transport events of all sizes, with the largest event being a landslide. In this example, it is the duration of individual sediment transport events (or autogenic processes) within a single segment that defines the temporal extent of correlation. However, when considering a STS with multiple, linked segments, the sediment efflux of one segment becomes the sediment influx to the next (e.g., sediment transport from a hillslope segment to a fluvial segment in a catchment). Therefore, in order for the sediment flux from a hillslope to be measured at the catchment outlet, it must propagate from the hillslope into the fluvial network. This means that rather than the absolute duration of individual sediment transport events defining the extent of correlation, we hypothesize that it is instead the time required to evacuate the sediment from the hillslope to a valley and ultimately into the fluvial system and hence be measured at the catchment outlet. This means that the (dis)connectivity of STS segments could influence the extent of correlation and the time scales of autogenic processes evident from a time series of sediment flux at the catchment outlet (*69*). For linked segments of a hillslope-fluvial system, the time scales of correlation would relate to the time required to remove and redistribute sediment from the hillslope to the fluvial network. If the hillslope-fluvial system is well connected, sediment delivered from the hillslope segment is fed directly into the fluvial segment; hence, we hypothesize that time required to evacuate sediment from the catchment is short, and hence, *T*_rw_ is short. This also means that the STS could convey sediment flux signals effectively through consecutive STS segments (*7*), depending on the autogenic processes and storage capacity of the STS segment in question (*65*). On the other hand, if a hillslope-fluvial system is disconnected, sediment is stored on the hillslope (*7*, *70*) where it is gradually removed and transported to the river network. This gradual liberation of sediment enhances the sediment flux exiting the catchment over long time scales (*70*). Many extremely large landslide deposits can remain in mountain landscapes for up to 10^4^ years (*54*), which contributes to sediment flux exiting the catchments over these time scales. This means that although the absolute landslide duration on the hillslope is short-lived, the time to evacuate the associated sediment from the catchment by fluvial processes is much longer, which we hypothesize will extend the time scales of temporal correlation (e.g., red noise) (*54*) and hence the time scales over which signals will experience shredding. From the above, it is evident that the interconnection of STS segments strongly influences the spectral geometry of influx at the outlet of the connected segments and controls how signals propagate between and through them [e.g., (*65*)].

We find that *T*_rw_ in the physical rice pile is independent of the rate of sediment supply; however, the behavior of smaller avalanche dynamics is not. The greater the sediment supply rate, the more frequent the occurrence of smaller avalanches in the rice pile (fig. S5); however, the frequency of the largest avalanches converges at the size of the largest system-scale event. Conversely, for natural and experimental STSs, this time scale is unlikely to be independent of the rate of sediment supply because topography can be built and filled faster with an increased sediment supply rate. For example, temporal correlation (red noise) in a cellular automata model of alluvial transport (*71*) extends up to a maximum time scale of river avulsion, and within a single deltaic system, the maximum autogenic time scale is denoted by a system-wide, lobe movement event and associated compensational filling of topography (*72*). In each case, the maximum autogenic time scale is akin to *T*_rw_ in the physical rice pile, but unlike *T*_rw_, they are dependent on the rate of sediment supply (*73*). Further work is needed to investigate the mechanisms that contribute to the longest duration autogenic events, which will define the autogenic time scales in both experimental and field-scale systems.

**Fig. 6.**
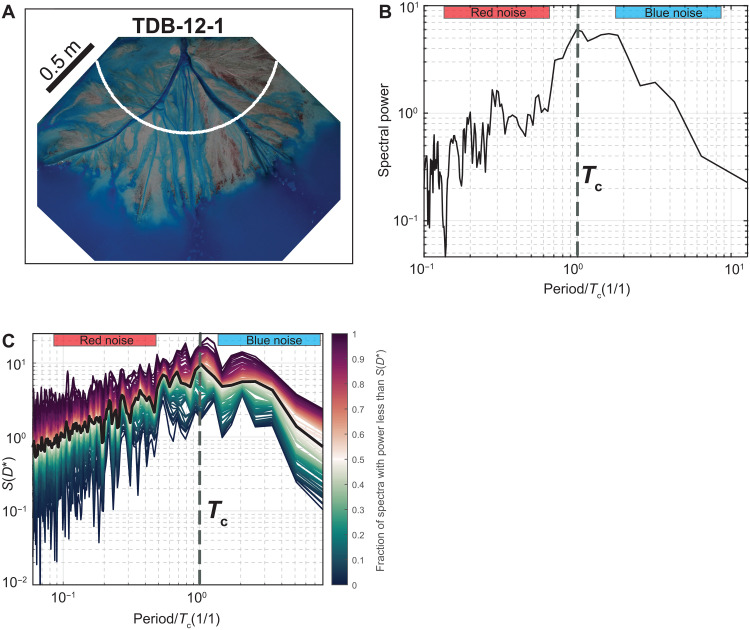
Surface morphology and power spectral analysis from delta basin experiment TDB-12-1. (**A**) Overhead photo from the delta basin experiment (*69*). Power spectra for preserved deposition rates were generated at every point (5-mm spacing) from the center portion of the radial white line, from which an average spectrum was generated. (**B**) Power spectra generated from a time series of sediment flux to the marine using the multi-taper method. Time is normalized by the compensation time scale (*T*_c_) (~49 hours). (**C**) Power spectra generated from a time series of preserved deposition rates (fig. S6) using the multi-taper method. The black line defines the ensemble average power spectra. Normalized by the long-term accommodation generation rate (0.25 mm/hour). Time is normalized by the compensation time scale (*T*_c_) (~49 hours).

Limitations of stratigraphic datasets (e.g., limited outcrop exposure, incompleteness and the assumption of linear sedimentation rate) make it difficult for field practitioners to explore details of autogenic processes over geological time scales. High-resolution time series of surface sediment fluxes and preserved deposition rates of an experimental delta run under constant boundary conditions (*74*) allow us to study the structure and time scales of autogenic processes in a system more analogous to that of field-scale systems, and one that contains morphodynamic behavior. We create power spectra of surface sediment flux from the terrestrial delta top to the marine, analogous to the efflux of rice from the rice pile (Fig. 6), which reveals temporal correlation (red noise) over all time scales up to the compensation time scale (*T*_c_) (*75*). *T*_c_ represents the truncation time scale of depositional processes in natural systems (*37*), analogous to *T*_wb_ that represents the largest autogenic event in the rice pile, and defines the maximum time scale of autogenic organization in stratigraphy (*66*, *75*). As with *T*_wb_ in the rice pile, the compensation time scale denotes a detectability time scale for signals within channelized systems, whereby input signal periodicity greater than *T_c_* are more likely to be recorded in both landscapes and strata (*6*, *33*, *46*). At time scales greater than *T_c_*, anticorrelation (blue noise) persists over all subsequent time scales. This spectral geometry is maintained within spectra generated from a time series of preserved deposition rates generated from the same experiment (Fig. 6).

Unlike the rice pile, power spectra generated from the experimental delta do not exhibit a white noise regime. Building on our understanding of physical rice pile processes and their contribution to autogenic spectral structure, we note that a white noise regime will not be present when the time scale of the longest correlated event (i.e., *T*_rw_) is equal to or exceeds the regeneration time scale (*T*_wb_). The convergence of these time scales in the power spectra of the experimental delta defines only one spectral rollover from red to blue noise. We hypothesize this to be because the duration of the maximum autogenic time scales (i.e., a system-wide, lobe movement event) and the compensation time scale [*T_c_*; approximately 49 hours for this experiment (*15*)] are of the same order of magnitude. Therefore, we emphasize that the spectra produced from a time series of landscape or stratigraphic measurables may not necessarily exhibit tripartite geometry, as this is dependent on system size and sediment supply rate which controls the relationship between *T*_rw_ and *T*_wb_ and so therefore the presence and duration of white noise. However, a long time series generated from landscape or stratigraphic measurables that is of sufficient temporal resolution should reveal both red and blue noise. The convergence of time scales could also happen in other geomorphic systems, such as landsliding in mountain catchments, whereby a tripartite spectral geometry would be prevalent when the reoccurrence interval of landslides exceeds the time needed to evacuate the landslide sediment from the catchment by fluvial processes. However, if the time to evacuate landslide sediment exceeds the reoccurrence interval, we expect this would result in a condensed spectral geometry showing one spectral rollover between red and blue noise (i.e., no white noise).

Within the rice pile *T*_rw_ and *T*_wb_ are linked by the maximum-size autogenic event, whereby *T*_rw_ represents the longest avalanche duration to evacuate this rice mass and *T*_wb_ represents the time required to regrade the rice-wedge with this same amount of mass. To investigate whether this parity of mass or volume might exist in close analogs of field-scale systems, we use volume fluxes from the experimental delta. Specifically, we calculate the volume of sediment exported between large-scale lobe movement events, representing the largest avalanche, to be approximately 0.030 m^3^, and the volume of sediment required to regrade delta topography by one channel depth, representing the regrading of the sediment wedge, to be approximately 0.024 m^3^. Those large-scale lobe movement events analyzed were limited to time scales less than *T*_c_ to enable the comparison, which the latter measurement of one channel depth across the delta is linked to the compensation time scale (*T_c_*), which is linked to the longer-term, wedge filling time scale (*T*_wb_).

### The severity of the signal shredder

Although autogenic processes within landscapes and strata show comparable temporal structure containing both red and blue noise, we have evidence that the shredding process may operate with differing severity. The spectral growth index (e.g., gradient of red noise) varies between the spectra, with the deposition rate power spectra following a much lower index value than the rice pile or surface delta fluxes (α = 0.8 versus α = 1.3 and α = 2.2, respectively).

Systems that evolve toward a critically self-organized state are defined as having spectral growth at 1/*f* (e.g., pink noise; α = 1) (*48*), where noise in the system is moderately correlated (*76*). We find that over short time scales, the surface delta fluxes have moderate correlation, with spectral growth at approximately 1/*f* (α = 1.3), whereas the rice pile has strong correlation, with spectral growth is approximately 1/*f*^2^ (e.g., red noise; α = 2) (*76*). Over these time scales, the strength in the correlation of the system indicates the frequency of erratic behavior away from the mean state; the stronger the correlation, the less frequent the chance of erratic behavior. To explain this, we refer to the dynamics present within a delta. When a channel network is stable on a delta top, the fluxes to the marine are consistent at high rates until the channel network collapses. The consistency of the channel network allows for the efficient propagation of sediment down system. However, during this period of stability, events such as infrequent breaches may occur that divert water and sediment to the delta top for short time periods but do not trigger an avulsion event. This is defined as erratic behavior, e.g., a rapid, temporary change in the system. This long-term stability intermixed with short term temporary fluctuations manifests as approximately 1/*f* noise in surface delta fluxes. However, we find that depositional fluxes have weak correlation, with spectral growth less than α = 1, indicating that the system has a considerable chance of being driven in a different direction at any time. Within the delta system, we hypothesize that the correlation in the system is defined by periods of no deposition, and hence during a period of stasis, the system will tend to remain in stasis up to a maximum time scale of *T*_c_. However, periods of deposition and erosion (over a range of spatiotemporal scales) interrupt periods of no deposition (e.g., long-term erratic behavior), which weakens the temporal correlation.

The differences in the strength of the correlation between surface and stratigraphic records could suggest variations in the intensity of the shredding process. Within pink noise (α ~ 1) and red noise (α ~ 2) systems, the stability of the sediment transport system could suggest that short-period signals propagate more effectively. While these signals would still experience some degradation, as they do not overwhelm autogenic fluctuations, their propagation is relatively uninterrupted. For example, when the channel is stabilized on a delta top, consistently high sediment flux rates allow for effective sediment transport down system. This would result in overall less degradation and possibly allow the imposed signal to be reconstructed by stacking records at the known periodicity. However, the erratic correlation present in systems where α < 1 (e.g., interruptions in sediment transport and deposition) suggests a stronger ability to smear signals through space and time. In these systems, the balance among deposition, erosion, and stasis is highly variable. Hence, the greater the lateral mobility of the system, the greater the chance of hiatuses and/or reworking of previously deposited sediment by erosion (e.g., the occurrence of long-term erratic behavior) and hence the lower the rate of spectral growth. This may result in a signal being completely obliterated (i.e., no returnable amplitude in a time series of sediment flux). If the severity of the degradation process does inversely scale with α, our results suggest that the depositional filter could act as a “super shredder” of environmental signals.

Signals with periodicity less than *T*_rw_ can sometimes be reconstructed from stacking landscape records, but this requires the record to be many multiples of the imposed periodicity, allowing the transport system noise to be averaged. Furthermore, signals with periodicity between *T*_rw_ and *T*_wb_ can be detected or obscured within a landscape depending on the amplitude of the signal. However, any signal detectable within landscape records may not be of sufficient periodicity or magnitude to overcome the longer, harsher stratigraphic shredding regime. Therefore, any resemblance of a signal would be completely removed within a time series of stratigraphic measurables (*4*, *6*).

### The nature of autogenic processes

The longest autogenic time scales evident in landscapes and strata (*T*_wb_ or *T*_c_) define rollovers to a blue noise regime within power spectra. Although the presence of this spectral regime is intuitive, it is rarely acknowledged in spectra generated from stratigraphic measurables; instead, spectra are described to be dominated by the presence of commonly known red and white noise (*68*, *77*). The lack of identification could be a result of how power spectra are commonly plotted; plotting power spectra as a function of frequency renders blue noise more difficult to identify than if plotted as a function of period. However, if blue noise is simply not present, this could result from the lack of availability of long time series datasets (either due to insufficient duration of the instrumental record or due to the availability of outcrop exposure), the incompleteness of the stratigraphic record favoring high-frequency fluctuations (*4*), the messy conversion of space to time from stratigraphic measurables (e.g., the assumption of linear sedimentation rates) (*78*), or the lack of dynamic equilibrium in STSs, generating nonstationary statistics and therefore rendering blue noise unobservable (*24*). The unknown presence of blue noise within power spectra generated from stratigraphic measurables has implications for generating statistical tests to detect the presence of environmental signals (*15*). The application of the autoregressive lag 1 model (*79*), typically for paleoclimatic studies, does not fully represent the spectral background structure generated by geomorphic variability (*33*), which could produce false positives and spurious signals. To ensure accurate detection of environmental signals, spectral estimations must provide a strong fit to the background structure. The potential universality of the presence of blue noise within the temporal structure of autogenic processes highlights the requirement to generate a statistical model to fit power spectra of this structure, which will allow the accurate detection of environmental signals over all autogenic time scales.

Overall, two key time scales emerge from the study of autogenic dynamics within a physical rice pile experiment which provide thresholds for both signal degradation (*T*_rw_: the event duration time scale) and enhanced signal detection (*T*_wb_: the system regrading time scale). The autogenic time scales presented provide a framework to predict the severity of signal shredding across Earth’s surface and to strata and establish robust confidence limits of signal detectability in landscapes and strata. We highlight the applicability of this framework to all segments of a sediment routing system (e.g., erosive catchments experiencing land sliding or fluxes to the deep marine) alongside systems that experience environmental stochasticity [e.g., earthquakes, storms, and floods; (*4*)].

## MATERIALS AND METHODS

### Experimental design

A suite of rice pile experiments were conducted in the Sediment Dynamics Laboratory at Tulane University to characterize the nature of the autogenic dynamics and assess the degree to which key autogenic time scales provide thresholds for signal shredding and detection.

The experimental apparatus is constructed of two vertical, parallel glass sheets 37.5 cm long, positioned 2.6 cm apart. Rice was fed (influx) to the pile from a dry particle feeder (Schenk Accurate) positioned 8 mm from the top surface, allowing a rice pile to form at a critical angle so that a dynamic topographic equilibrium was achieved. Over the suite of experiments, influx was defined between a minimum and maximum range (0 and 0.78 g s^−1^), controlled at 1-s intervals via a computer connected to the sediment feeder, which directly feeds the pile. Efflux was measured at approximately 1-s intervals using an Ohaus EX12002 balance (accuracy and precision of 0.1 g). The balance has a maximum mass of 12 kg, and all experiments were run until the balance was saturated. The dimensions of rice grains used in the experiments have a diameter of 2.5 ± 0.5 mm, a length of 8 ± 0.5 mm, and a mass of 0.02 g (table S2). The experimental setup used here is similar to that of the physical rice pile of Frette *et al.* (*49*)

To ensure that the efflux data are driven only by the internal autogenic dynamics of the rice pile and not triggered by external noise, we analyzed accelerations within the room when the sediment feeder was on and off, when sediment feeder was on but with no rice delivery, and when rice was delivered (fig. S7). Accelerations were measured using the Phyphox application on an iPad, which records *x*, *y*, and *z* accelerations at an increment of ~0.05 s to two significant digits of acceleration with SI units. The raw acceleration data, alongside the power spectra of the time series, were analyzed to confirm that external vibrations were not triggering avalanches or that external vibrations did not occur at repeating frequencies.

A series of experiments were conducted where rice was fed directly from the sediment feeder to the scale to confirm we had high temporal control over the influx rates and cycles imposed. We generated power spectra from the time series, which confirms that white noise was present across all frequencies, except a spike in power if periodicity was imposed (fig. S8).

First, a control experiment was run for 9 hours with a constant influx rate of 0.37 g s^−1^. The influx rate denotes the mean rate of the sediment feeder, and experimental run time was defined by the time to saturate the balance at the defined influx rate. This experiment was used to define the full spectral structure generated by a physical rice pile and quantify autogenic time scales evident from rollovers between spectral regions. Using this baseline behavior, a suite of nine experiments (table S1) was used to explore the influence of influx rate on the autogenic dynamics and time scales found in the control experiment. These experiments varied systematically in intervals of approximately 0.1 g s^−1^ from the minimum to the maximum influx rates available on the sediment feeder.

To explore limits of signal shredding and signal detection, a matrix of 36 experiments was run with cyclic influx of different periods and amplitudes. To achieve parity with the control experiment, a mean influx rate of 0.37 g s^−1^ was attained for all cyclic experiments. Nine periodicities were chosen to cover the range of autogenic time scales evident in the control experiment: 6, 12, 24, 48, 100, 250, 500, 1000, and 2000 s. The amplitude of the cycles was chosen as percentages of the mean feed rate (0.37 g s^−1^), increasing in 25% intervals from 25% (0.0925 g s^−1^) to 100% (0.37 g s^−1^).

### Signal detection from power spectra

Discrete time-power spectral densities of efflux time series (power spectra) were generated using the multi-taper method with two tapers. Key autogenic time scales can be observed by eye on the power spectra as “rollovers or “gradient breaks.” To delimit these time scales accurately, we use the “findchangepts” function in MATLAB. This function is controlled by two key input parameters: the maximum number of changes and the type of change to detect (e.g., variations in mean, SD, and gradient). For our spectra, we specify two changes (to account for the presence of two rollovers in the spectra) and use linear as the type of change to detect, applied on log-transformed spectral data. This method detects changes in the mean and slope of the input spectra, which can be inverse log-transformed to solve for the power-law exponent of the fit.

To make a statistical statement about the presence or not of an influx signal in the power spectra, a confidence band for the discrete time-power spectral densities is required. Using 25 realizations of the control experiment, we generated power spectra for each realization. For each periodicity, we rank the associated power values from all 25 spectra into ascending order to calculate the percentage of the realizations that fall above a given power for each period. From this, we calculate an estimate of the 95th percentile confidence band.

### Signal degradation

To quantify the amount of degradation a signal experiences during propagation, we stack the efflux time series into lengths equal to the input period and take the mean of the efflux for each second over the imposed periodicity. From this, we gain a mean ensemble efflux to which we fit a sine wave with a period equal to the known input and are returned an amplitude and phase based on the signal present in the mean ensemble efflux. We compare the amplitude of the signal evident in the ensemble efflux to that of the known input signal and quantify a percentage similarity (fig. S2).
