# Supplementary material for: The degradation and detection of environmental signals in sediment transport systems
Source: Sci Adv. 2023 Nov 3;9(44):eadi8046. doi: 10.1126/sciadv.adi8046 (PMC10624340; doi:10.1126/sciadv.adi8046)
Supplement: Supplementary file 1 — Figs. S1 to S8 Tables S1 and S2 [file sciadv.adi8046_sm.pdf]

Science Advances
AAAS

Supplementary Materials for

The degradation and detection of environmental signals in sediment transport systems

Chloe Griffin *et al.*

Corresponding author: Chloe Griffin, c.l.c.griffin@liverpool.ac.uk

Sci. Adv. **9**, eadi8046 (2023)
DOI: 10.1126/sciadv.adi8046

This PDF file includes:

Figs. S1 to S8
Tables S1 and S2

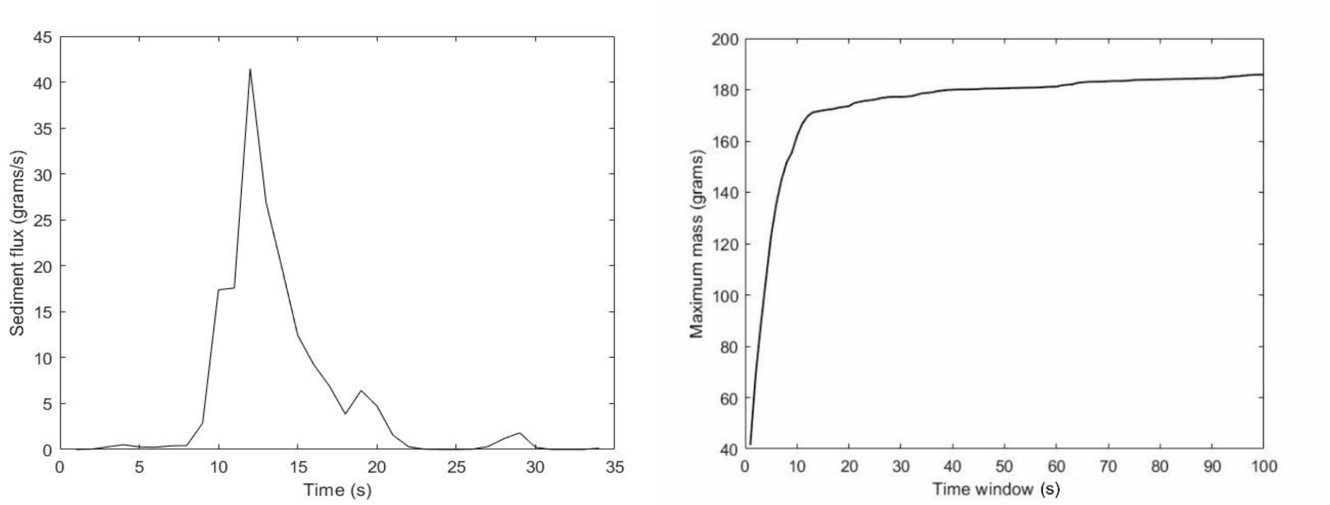

Fig. S1.

Calculation of M_{max}.

Left: Time series of efflux over the duration of the largest avalanche in the rice pile. Right: Maximum mass effluxed over increasing time windows of observation, where at timescales greater than T_{rw}, maximum mass plateaus

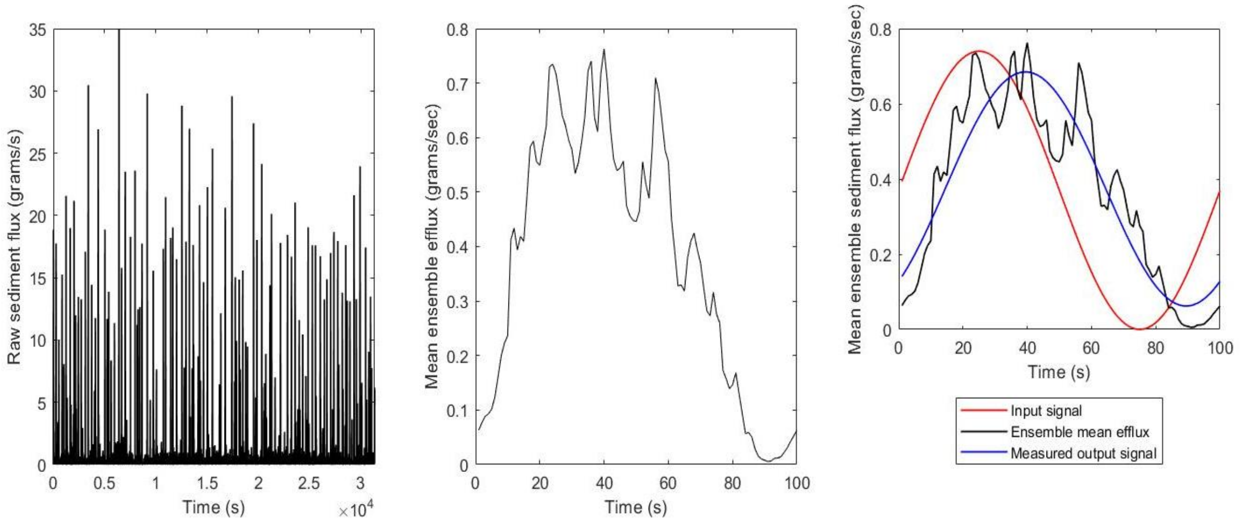

Fig. S2.

Methodology to determine the amount of degradation a signal has experienced during propagation.

The raw efflux time series is generated for an experiment with imposed cyclicity (here, period 100s, amplitude 0.37 grams/sec). Then, the efflux is divided into lengths equal to the period of the input signal and the mean efflux over each second of the imposed periodicity is calculated. After this, a sine wave is fitted to the stacked data, where the periodicity is defined and the amplitude and phase is calculated based on the individual dataset. The amplitude of the output signal is then compared to the amplitude of the known input signal to calculate percentage similarity.

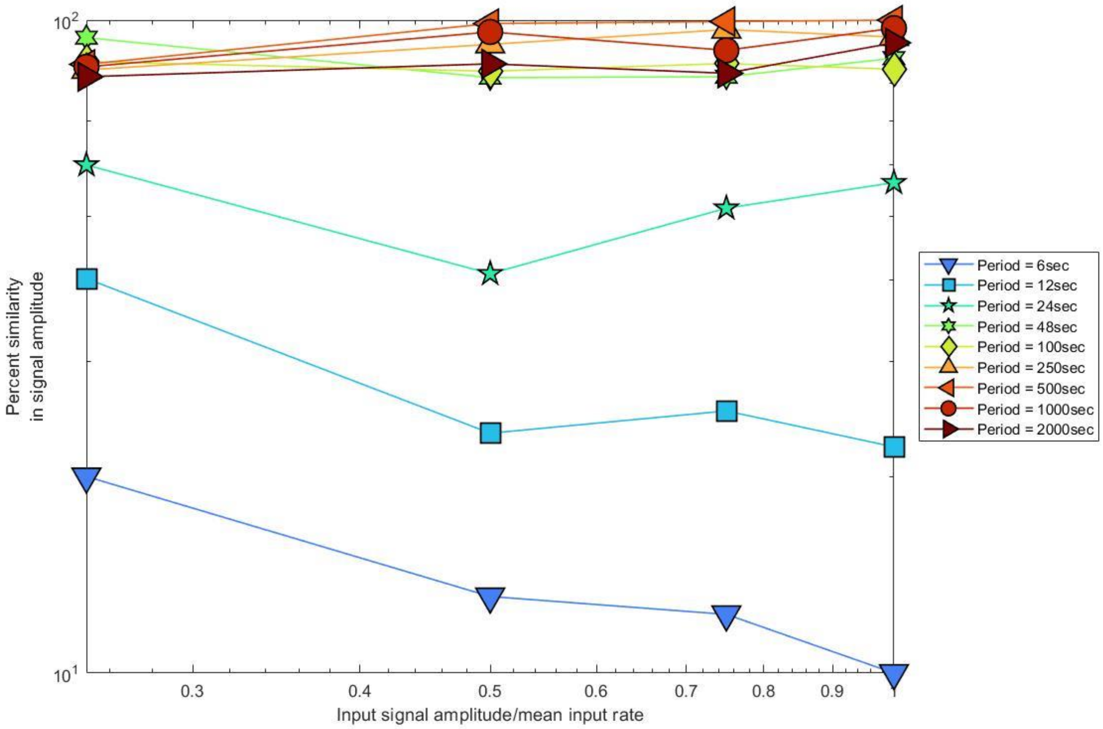

Fig. S3.

Signal degradation as a function of both input period and amplitude.

Signals with periodicity below T_{rw} experience severe degradation, whereas signals with periodicity greater than T_{rw} experience minimal degradation.

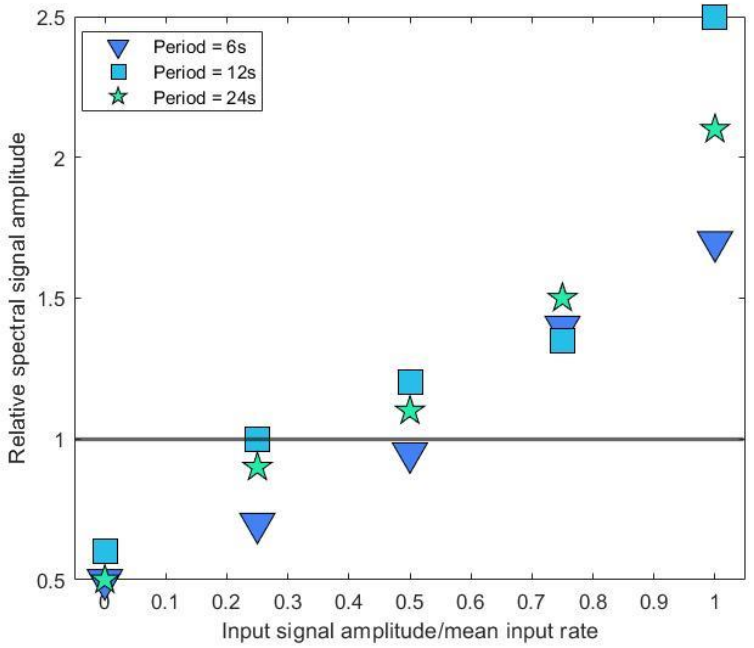

Fig. S4.

Detectability of square wave input signals with periodicity less than T_{rw} and with an amplitude equal to or below the mean feed rate.

Power of the signal spike at the imposed periodicity compared to the power of the 95% confidence band at the imposed periodicity. The data at 0 amplitude represents an experimental run with no imposed periodicity. Y-axis data points are calculated as power at imposed period/power of confidence band at imposed period, hence values greater than 1 breech the confidence band.

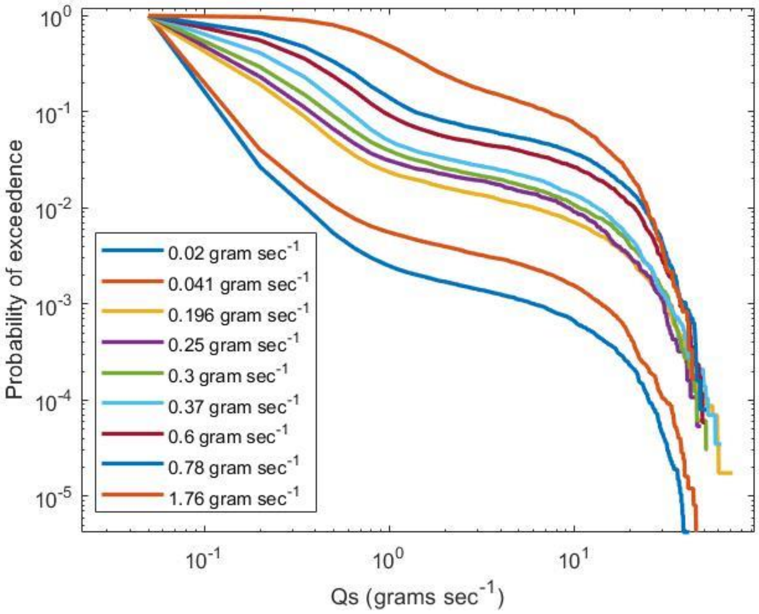

Fig. S5.

Distribution of avalanche sizes within the efflux time series from nine experiments with increasing influx rate.

All the time series show a heavy-tailed distribution, however as the influx rate increases, there is an increased probability of a certain sized event occurring, but the distributions converge at the largest event.

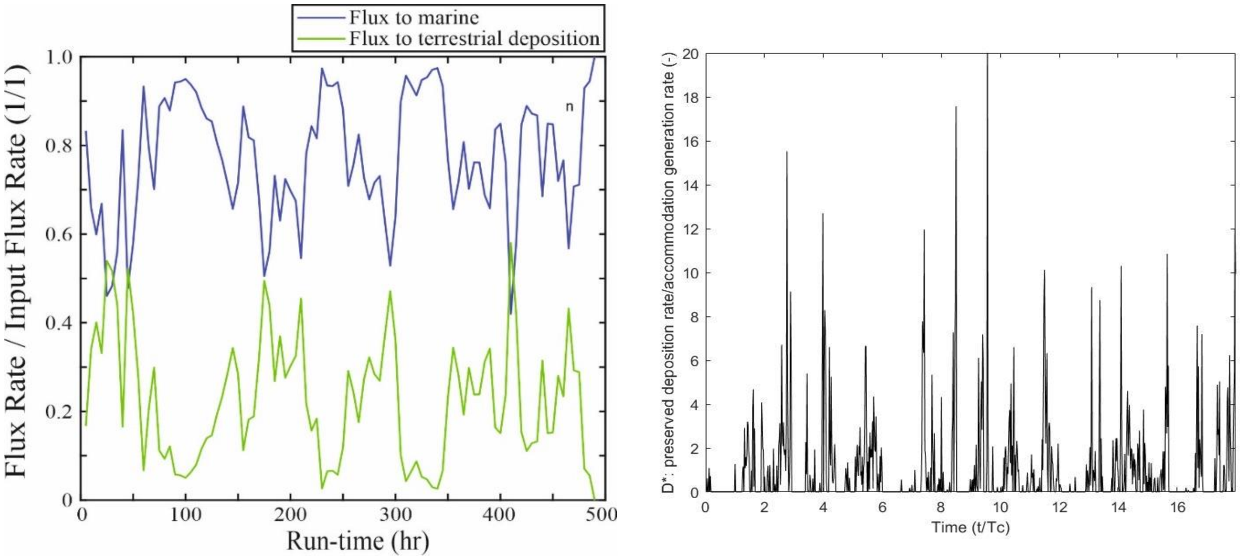

Fig. S6. Time series of surface and stratigraphic dynamics from the delta basin Left: Time series of sediment flux to the marine realm, versus sediment flux to the terrestrial deposit within the experimental delta basin. The time series is generated from experiment TDB-12-1 (*71, 77*). The time series of sediment flux to the marine was generated as follows. Topographic maps were taken over the duration of the experiment. Successive topographic maps were differenced to generate an isopach map (map of sediment thickness). Using the measured and imposed sea level each hour, all pixels in the terrestrial realm on the isopach map were summed, and this was multiplied by the x and y node spacing on the map to get a bulk volume of sediment deposit in the terrestrial over this time period. Then, a bulk sediment flux to terrestrial deposition was calculated by dividing by the time between the maps, and then converted to a volumetric sediment flux to terrestrial deposition by multiplying the bulk sediment flux by the fraction of the deposit which is sediment (1-porosity). This has been previously measured as 0.5 with the same mixture of sediment. To get a mass flux to terrestrial deposition, the volumetric flux was multiplied by sediment density (2650 kg/m^3). The mass flux to terrestrial deposition was then subtracted from the known mass input flux (1.41 kg/ hour) to get the flux to the marine. Right: Time series of preserved deposition rates measured from data points spaced 5mm apart along a radial arc within the experimental delta basin.

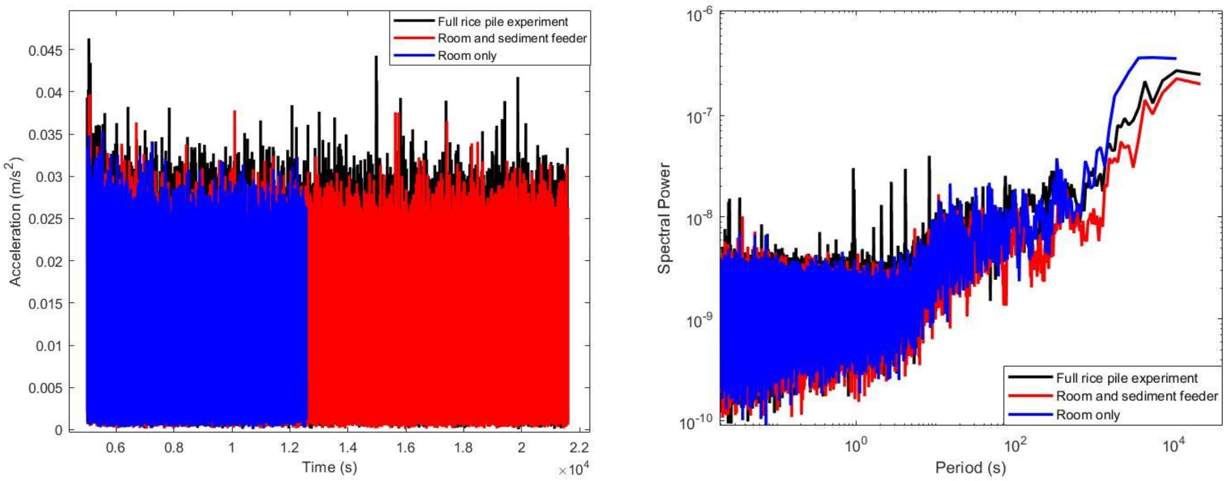

Fig. S7.

Acceleration analysis to ensure the dynamics evident are inherent to the rice pile.

Left: Raw acceleration time series for the room only, the room and sediment feeder with no rice pile, and then over the duration of a full experiment. Right: Power spectra (generated using the MTM method with 2 tapers) of the acceleration time series for all three scenarios. Neither the raw time series or power spectra show evidence of external noise occurring at repeating frequencies

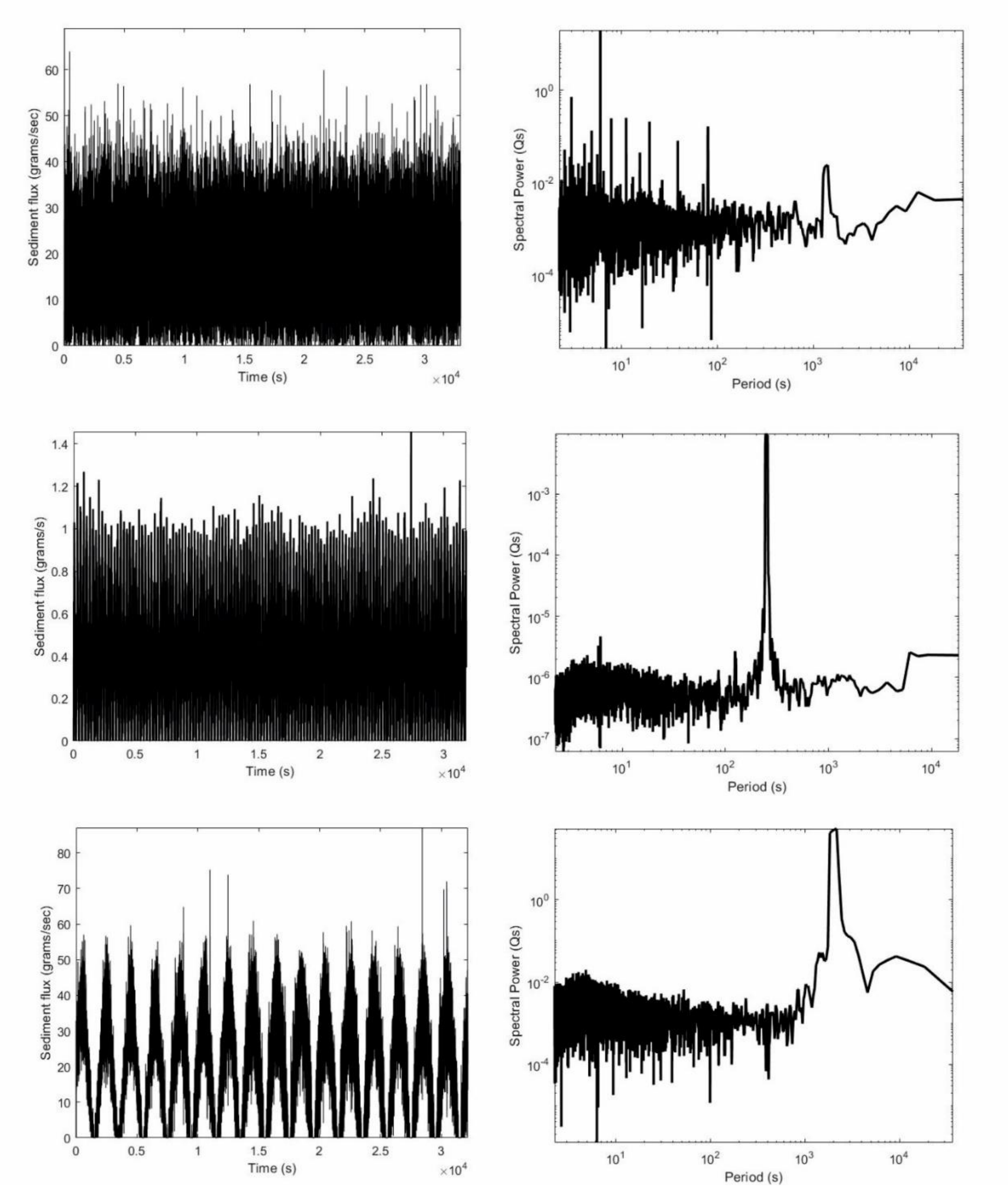

Fig. S8.

Time series and power spectra from three calibration experiments where rice was fed directly from the sediment feeder to the scale.

Top: Cyclic experiment (periodicity 6s, amplitude 0.37 gram sec^{-1}) with a signal evident at 6s. Middle: Cyclic experiment (periodicity 250, amplitude 0.37 gram sec^{-1}) with a signal evident at 250s. Bottom: Cyclic experiment (periodicity 2000s, amplitude 0.37 gram sec^{-1}) with a signal evident at 2000s.

Table S1.

Supply characteristics for the individual rice pile experiments, both constant and cyclic feed rates

.

Experiment	Stage	Desired mean feed rate (grams/sec)	Actual mean feed rate (grams/sec)	Period of forcing (s)	Amplitude of forcing (gram/sec)
1	Control	0.37	0.358	-	-
2.1	Steady	0.02	0.027	-	-
2.2	Steady	0.041	0.052	-	-
2.3	Steady	0.196	0.22	-	-
2.4	Steady	0.25	0.28	-	-
2.5	Steady	0.3	0.29	-	-
2.6	Steady	0.6	0.73	-	-
2.7	Steady	0.78	0.99	-	-
2.8	Steady	1.76	2.1	-	-
3.1	Cyclic	0.37	0.358	6	0.37
3.2	Cyclic	0.37	0.358	6	0.2826
3.3	Cyclic	0.37	0.358	6	0.185
3.4	Cyclic	0.37	0.358	6	0.0925
3.5	Cyclic	0.37	0.358	12	0.37
3.6	Cyclic	0.37	0.358	12	0.2826
3.7	Cyclic	0.37	0.358	12	0.185
3.8	Cyclic	0.37	0.358	12	0.0925
3.9	Cyclic	0.37	0.358	24	0.37
3.10	Cyclic	0.37	0.358	24	0.2826
3.11	Cyclic	0.37	0.358	24	0.185
3.12	Cyclic	0.37	0.358	24	0.0925
3.13	Cyclic	0.37	0.358	48	0.37
3.14	Cyclic	0.37	0.358	48	0.2826
3.15	Cyclic	0.37	0.358	48	0.185
3.16	Cyclic	0.37	0.358	48	0.0925
3.17	Cyclic	0.37	0.358	250	0.37
3.18	Cyclic	0.37	0.358	250	0.2826
3.19	Cyclic	0.37	0.358	250	0.185
3.2	Cyclic	0.37	0.358	250	0.0925
3.21	Cyclic	0.37	0.358	500	0.37
3.22	Cyclic	0.37	0.358	500	0.2826
3.23	Cyclic	0.37	0.358	500	0.185
3.24	Cyclic	0.37	0.358	500	0.0925
3.25	Cyclic	0.37	0.358	1000	0.37
3.26	Cyclic	0.37	0.358	1000	0.2826

3.27	Cyclic	0.37	0.358	1000	0.185
3.28	Cyclic	0.37	0.358	1000	0.0925
3.29	Cyclic	0.37	0.358	2000	0.37
3.3	Cyclic	0.37	0.358	2000	0.2826
3.1	Cyclic	0.37	0.358	2000	0.185
3.2	Cyclic	0.37	0.358	2000	0.0925

Table S2.
Characteristics of the rice used in the experiments.

Name	Par Excellence® Premium Brown Rice
Description	Long grain, parboiled
Length	8 ± 0.5mm
Width	2.5 ± 0.5mm
Aspect ratio	3.2
Average mass	0.195g
No. density	0.78 ± 0.1 g/cm^3
Angle of repose	45-47°
